# Supplementary material for: A whole-animal phenotypic drug screen identifies suppressors of atherogenic lipoproteins
Source: eLife. 2026 Aug 27;14:RP105314. doi: 10.7554/eLife.105314 (PMC13521566; doi:10.7554/eLife.105314)
Supplement: Supplementary file 2 [file elife-105314-code1.zip › JHDL_primary_result/PIMETHIXENE MALEATE/PIMETHIXENE MALEATE_JHDL_7_039_009_2019_06_14_11_21.pdf]

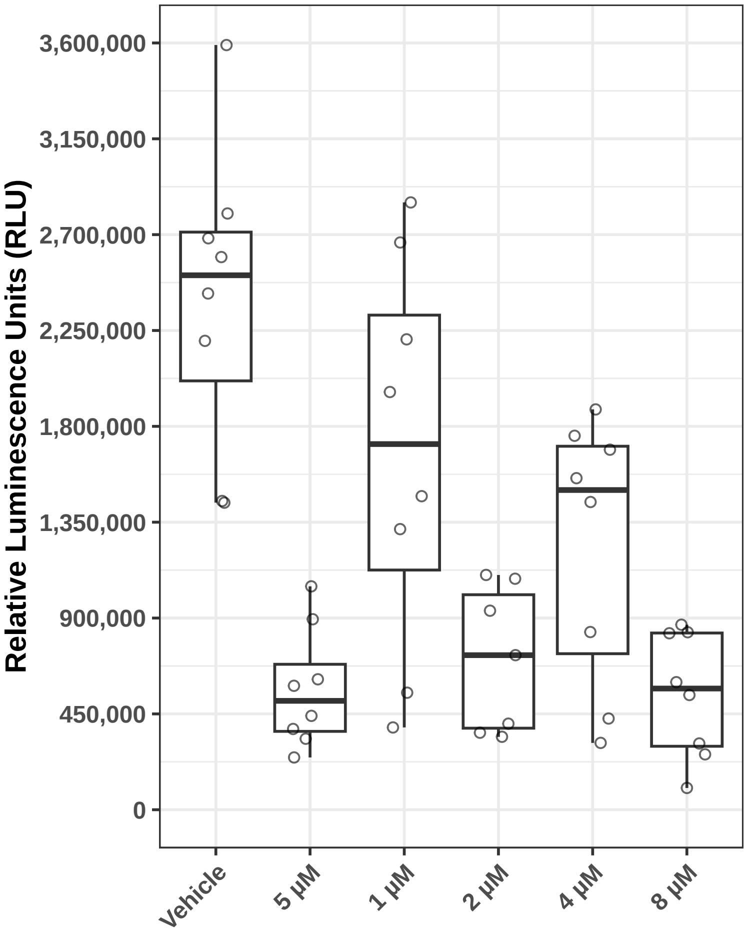

Relative Luminescence Units (RLU)
3,600,000
3,150,000
2,700,000
2,250,000
1,800,000
1,350,000
900,000
450,000
0
Vehicle
5 μM
1 μM
2 μM
4 μM
8 μM
